# Callogenesis and cell suspension establishment of tropical highland blackberry (*Rubus adenotrichos* Schltdl.) and its microscopic analysis

**DOI:** 10.1186/s40064-016-3381-0

**Published:** 2016-10-05

**Authors:** Alexander Schmidt-Durán, Carlos Alvarado-Ulloa, Randall Chacón-Cerdas, Luis Fernando Alvarado-Marchena, Dora Flores-Mora

**Affiliations:** 1 Centro de Investigación en Biotecnología of the Escuela de Biología, Instituto Tecnológico de Costa Rica, Cartago, Costa Rica; 2 Laboratorio Institucional de Microscopía, Instituto Tecnológico de Costa Rica, Cartago, Costa Rica

**Keywords:** *Rubus adenotrichos*, Callogenesis, Cell suspensions, Antioxidants, Cell differentiation

## Abstract

Blackberries are fruits produced worldwide, with 25 % of their production centered in Mexico, Central and South America. Tropical highland blackberry is a fruit that can potentially enhance human health, due to their high content in phenolic compounds, which include anthocyanins, phenolic acids, tannins (gallotannins and elagitannins) and flavonoids. Therefore, the overall aim of this study is the development of a callus induction protocol, the establishment of blackberry cell suspensions (*Rubus adenotrichos* Schltdl.) and their cell analysis through optical microscopy and TEM, for the potential production of phenolic compounds. In order to produce callogenesis, segments of blackberry leaves were disinfected and placed in different concentrations of 2,4-D and the control media (0; 0.5; 1.0; 1.5; 2.0; 2.5 and 3.0 mg/l of 2,4-D); obtaining the higher size of calli in the medium with 1.5 mg/l of 2,4-D. After this determination, and for this specific treatment, a growth curve was performed through the use of fresh and dry weight parameters, in order to identify each of the growth stages. Furthermore, the calli obtained from the 1.5 mg/l of 2,4-D treatment were placed in two different culture media (MS and MS supplemented with 1.5 mg/l of 2,4-D) in order to establish the cell suspensions and the growth curve. To the best treatment, the total polyphenols were also quantified. It was determined that the MS medium is ideal for the growth and disintegration of the cell suspensions, obtaining 0.0256 mg of gallic acid/g of fresh sample. Finally, a cell callus and cell suspension analysis was performed through OM and TEM, evidencing a higher hystological differentiation in the calli, as well as the observation of antioxidant storage in the plastids.

## Background

Higher plants constitute the source of a wide range of biochemical compounds that include natural pigments, flavors, fragrances, pesticides, pharmaceuticals and food additives; all of these compounds are classified as secondary metabolites (Farjaminezhad et al. 2013; Chattopadhyay et al. 2002). Nowadays, there is an increasing interest in antioxidants, particularly those which prevent the negative effects of free radicals in the human body, with a considerable higher preference in natural antioxidants rather than those from synthetic origin (Abdalla and Roozen 1999).

The consumption of plant products such as fruits and vegetables rich in antioxidants provide benefits to human health (Cerón et al. 2010); mainly in reducing the risk of cardiovascular diseases (Wang et al. 2011; Franzini et al. 2010; Sadani and Nadkarni 1996), diabetes (Ramful et al. 2010; McCune and Johns 2002), hypercholesterolemia (Mateos et al. 2005), and insulin resistance (Egan et al. 2001). Additionally, its aids in the prevention of other illnesses such as cancer (Ezzedine et al. 2010; Rossi et al. 2008; Collins 2005), arthritis, arteriosclerosis (Jaswal et al. 2003), brain dysfunction and reduces processes related to accelerated aging (Salmon et al. 2010; Bonetto et al. 2009).

Among the different fruits, genotypes and crops, there is a considerable difference in the quantities and types of phenolic antioxidants and their conjugates that can be found (Azofeifa et al. 2013; Cerón et al. 2010 *)*. Berries have been denominated as the fruits which can potentially enhance human health due to their high phenolic compound content. Most of the biological active compounds include polyphenols, such as anthocyanins, phenolic acids, tannins (gallic and elagitannins), flavonoids, carotenoids, and vitamin C (Azofeifa et al. 2013).

The *Rubus* genre is found worldwide (Azofeifa et al. 2013), with 25 % of the total world production focused on Mexico, Central America and South America, and being a crop of economic importance in these regions (Acosta-Montoya et al. 2010). This crop constitutes an important source of compounds of interest, such as secondary metabolites with antioxidant effect, which can be applied to reducing risks associated to certain diseases (Mertz et al. 2007).

The fruit of the *Rubus adenotrichos* Schltdl. species is commonly known as blackberry or brambleberry and its habitat is found in the mountain regions of Mexico, Ecuador and Costa Rica (Martínez-Cruz et al. 2011). The main polyphenols that are comprised in the *Rubus adenotrichos* Schltdl. blackberry are cyanidin 3-glucoside, cyanidin-3-malonyl glucoside, lambertianin C and sanguiin H-6 (Azofeifa et al. 2013). Recent research have determined that this species originated from Costa Rica, and presents high antioxidant contents (Acosta-Montoya et al. 2010; Mertz et al. 2009). Moreover, the protective effect of blackberry (*Rubus adenotrichos* Schltdl.) juice has been established against human epidermal cell, as well as in a reconstructed model of skin damaged by UVB radiation (Calvo-Castro et al. 2014). However, all the studies have been performed with the fruit or the juice, and the production of these compounds through biotechnological techniques has not been explored.

The plant cell culture techniques provide an appealing alternative for the production of valuable secondary metabolites, and they have been used throughout the years as a tool for the elucidation of the metabolites biosynthesis (Farjaminezhad et al. 2013). The first step to follow in this process corresponds to the callus induction, which consists of a mass of non-differentiated cells. From an engineering perspective, cell suspensions have a higher potential for industrial applications, when compared to the plant tissue and organ culture. A cell suspension is obtained through the transfer of a portion of a relatively friable plant callus into a liquid culture medium, while maintaining the correct aeration, agitation, light, and temperature conditions, as well as other physical parameters (Chattopadhyay et al. 2002).

The overall objective of this study is to develop protocol for callus induction and cell suspensions of blackberries (*Rubus adenotrichos* Schltdl.), intended for antioxidant production as well as their cell analysis.

## Methods

### Callogenesis induction

Complete, young leaves from plagiotropic axes located in the second and third position from the apex, were taken from red thorn blackberry plants (*Rubus adenotrichos* Schltdl.); derived from a greenhouse and which presented a previous eight day-application of Agrimycin^®^ and Benomyl^®^ (5 g/l, in both cases) and an application of Butrol^®^ (0.175 ml/l) 2 days before performing the introduction.

The leaves were transferred to the laboratory, where they were cleansed with water and liquid detergent during 10 min; later they were placed for 30 min in Benomyl^®^ (3 g/l), followed by the execution of a preliminary cut of the tissue into rectangular segments which included the main leaf vein. After this, the segments were introduced in the laminar flow hood, and a disinfection process was performed with the use of 1 % NaOCl for 8 min and 0.5 % NaOCl for 10 min, by rinsing three times with sterile distilled water between each disinfection. The leaf segments were reduced to a size of 1.0 cm^2^, including the main leaf vein and they were placed in a 1000 ppm ascorbic acid solution for 10 min.

With the prepared plant material, six callus induction treatments were established, in a Murashige and Skoog (MS) (1962) culture medium with 0.5; 1.0; 1.5; 2.0; 2.5 and 3.0 mg/l of 2,4-D and a control for the treatments. The callus induction was performed during 60 days and at 21 ± 2 °C, performing 30 repetitions per treatment. After this, the fresh and dry weight for each treatment was determined, and an analysis of variance (ANOVA) (α = 0.05) was performed for the analysis of the results obtained, using the Minitab 16 (2011) statistical software, the best treatment was used in order to trace the callus growth curve for the best treatment. To execute this, samples consisting of 1.0 g of callus in the multiplication stage were used and placed in the same callus induction medium that was selected as the best treatment. Every 3 days, three flasks with callus were randomly selected, for the determination of fresh and dry weight, during 42 days.

### Establishment of the cell suspensions and total polyphenol quantification

Through the selection of the best callogenesis treatment obtained, it was possible to proceed with the establishment of the cell suspensions. Therefore, 2 g of callus grown in semi-solid medium were transferred in each of the 250 ml erlenmeyer flasks prepared with 50 ml of liquid culture medium. Two different liquid culture mediums were used: MS medium without growth promoters and the best callogenesis treatment. Five flasks were inoculated for each type of culture medium, using a 100 rpm agitation and they were placed in the dark at 21 ± 2 °C, during a week.

In order to obtain fine plant cell suspensions, they were filtered through the use of a sterile 100 mesh sieve and a concentration of 1.0 × 10^4^ cell/ml were placed in each flask, establishing 15 repetitions for each treatment. Two cell counts were performed weekly, though the use of a light microscope during 38 days, in order to establish the total cell count. The data obtained was used to generate a growth curve for the cell suspensions per treatment.

Finally, a total polyphenol quantification was performed for the best treatment at the Centro Nacional de Ciencia y Tecnología de Alimentos (CITA) of the Universidad de Costa Rica, through spectrophotometry method to determine the equivalent mg of gallic acid present.

### Cell analysis through OM and TEM

The analysis of blackberry (*Rubus adenotrichos*) cells obtained from the best callus treatment and the best cell suspension treatment was performed in order to evaluate the hystological differences between cells from calli and suspensions. To execute this analysis, a callus sample and 0.5 ml of cell suspension were observed through an optical microscope (OM). The callus samples observed presented a reddish-purple coloration, due to the intracellular storage of compounds as a response to light exposure.

For the visualization under the transmission electron microscope (TEM), segments of callus of approximately 0.5 cm^2^ and 15 ml of a previously centrifuged cell suspension were processed. After this, the standard biological sample procedure reported by McDonald (2014) and Hayat (2000) was followed, which consisted of the fixation of the sample for 2 days at 4 °C in 2 % glutaraldehyde and paraformaldehyde dissolved in 0.15 M phosphate buffer, at a pH of 7.4, afterwards a post-fixation in 1 % osmium tetroxide was executed. Next, the samples were dehydrated in gradual dilutions of acetone, and they were polymerized in low viscosity resin (Spurr^®^). The ultrathin cuts were dyed with 2 % uranyl acetate during 15 min and then viewed through the Jeol JEM 2010 electronic microscope.

## Results

### Callogenesis induction

In the case of fresh and dry weight, the data presented variance randomness, homoscedasticity, and homogeneity, after its conversion through Johnson transformation (p > 0.05). In Table 1, it is possible to observe that most treatments presented values above 90 % of explants with callus formation, except for the culture medium with 1.0 mg/l of 2,4-D. The medium with 1.5 mg/l of 2,4-D presented the highest callogenesis percentage, and it was also observed that, for fresh and dry weight, it was the treatment that evidenced the highest biomass values. Therefore, this medium was selected as optimal for the callogenesis induction for this variety of blackberry plants. The transformation equation of the data for fresh weight was of 1.44165 + 0.435073 * Ln [(X − 0.494678)/(1823.51 − X)], while the equation for dry weight was 1.34482 + 0.494713 * Ln [(X − 0.0560235)/(138.416 − X)].

**Table 1:** Callogenesis percentage and ANOVA for fresh and dry weight of the callogenesis induction treatments

Treatment (mg/l de 2,4-D)	Callogenesis percentage (%)	Fresh weight*	Dry weight*
0.5	93.55	61.86 c	7.31 b
1.0	70.73	93.93 bc	9.14 b
1.5	97.06	382.94 a	36.07 a
2.0	93.55	324.53 ab	29.36 ab
2.5	94.12	18,694 abc	17.83 ab
3.0	93.75	354.17 abc	31.40 ab

* The different letters indicate significant statistical differences

**Fig. 1:**
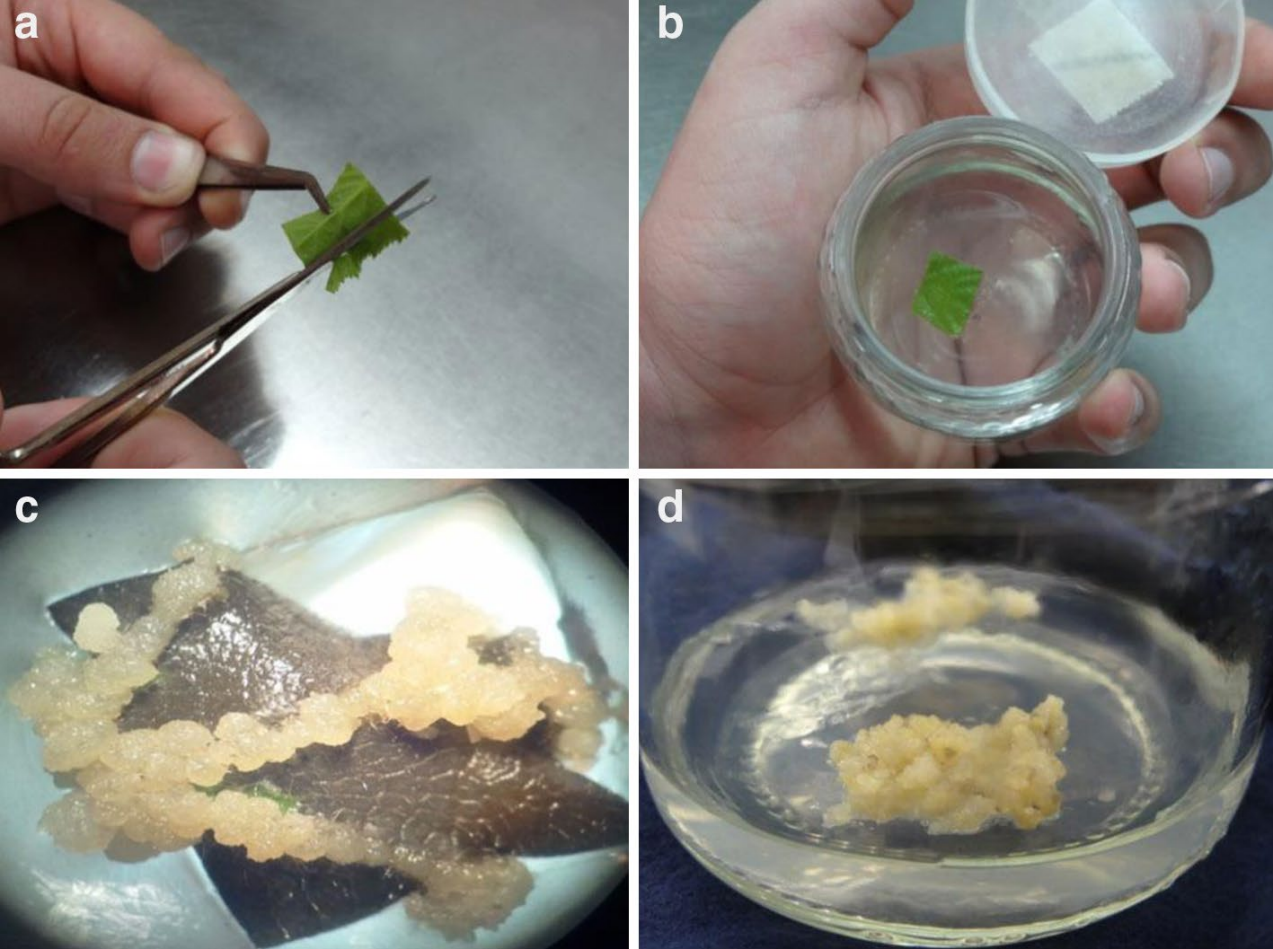
Blackberry (*Rubus adenotrichos* Schltdl.) callogenesis process, where an initial cut is performed in the leaf tissue (**a**), then the leaf segment is placed in the MS culture medium with 1.5 mg/l of 2,4-D (**b**); the development of callus occurs after 60 days (**c**) and the callus propagation (**d**)

**Fig. 2:**
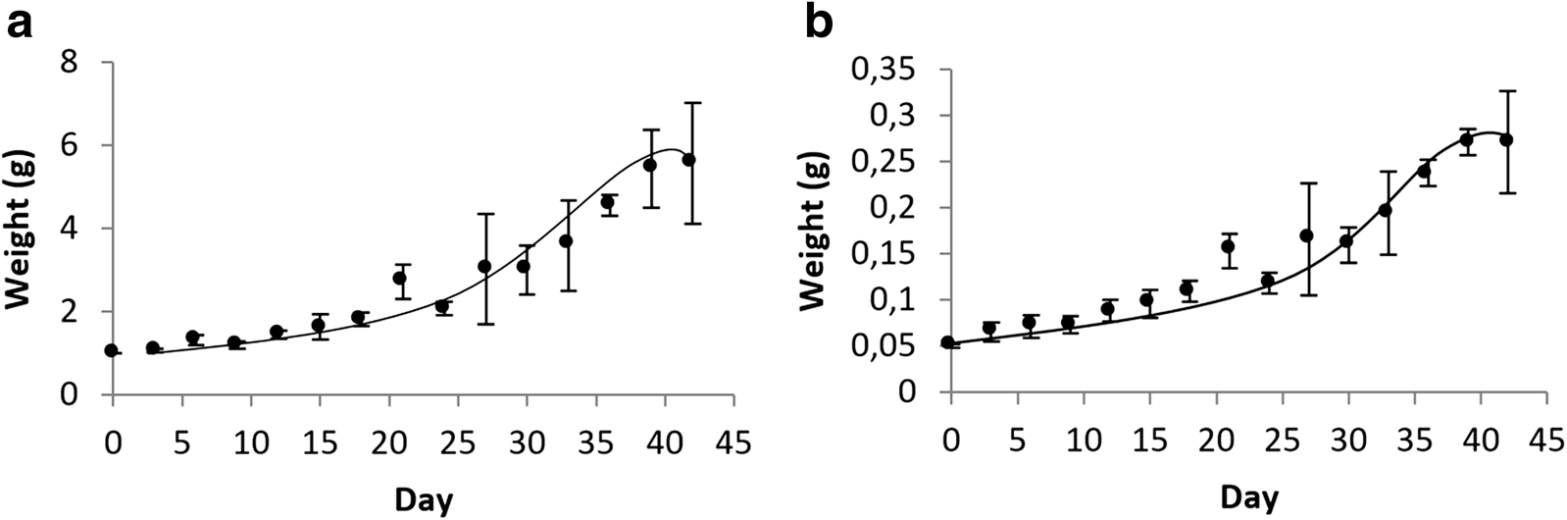
Blackberry (*Rubus adenotrichos* Schltdl.) growth curve for fresh weight (**a**) and dry weight (**b**) of the culture medium treatment with 1.5 mg/l of 2,4-D

In Fig. 1, it is possible to observe the callogenesis process of the treatment with 1.5 mg/l of 2,4-D. After the disinfection, a cut to the foliar segment is performed to favor the callus formation (Fig. 1a). It can be observed that the callus is formed in the abaxial face of the leaf, in the plant vessels and where the cuts were performed (Fig. 2b). This initial callus is used for the propagation and determination of the growth curve.

In Fig. 2 it is possible to observe the growth curves of the calli, in fresh and dry weight, of the 1.5 mg/l de 2,4-D treatment. Figure 2 presents a growth curve, which, on average, evidenced a callus growth from 1 g to approximately 5.5 g, presenting a latency phase (lag phase) of approximately 9 days, and reaching the stationary phase until the 39th day of cell growth. Additionally, it is possible to observe the growth curve obtained with the dry weight data. The R_2_ found by correlating both variables is of 99.22 %.

### Establishment of the cell suspensions and total polyphenol quantification

**Fig. 3:**
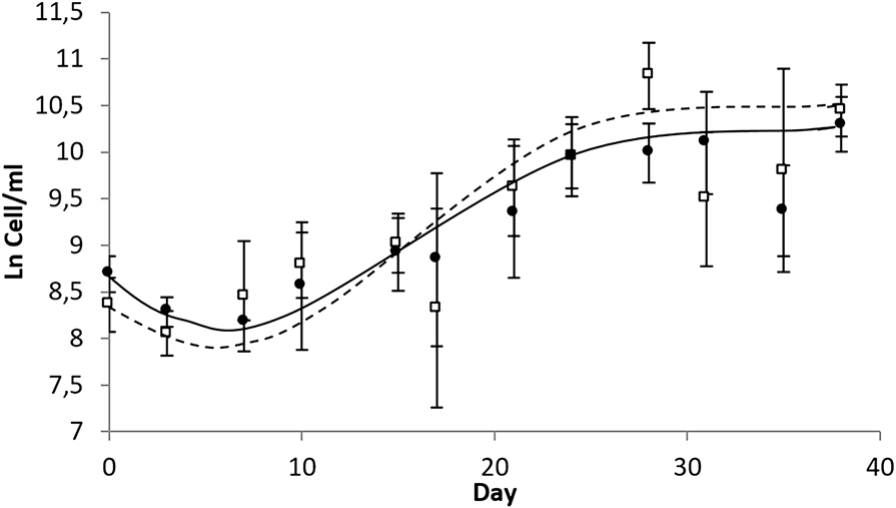
Comparison of the growth curves for the blackberry (*Rubus adenotrichos* Schltdl.) plant cell suspensions, grown in MS medium (*filled circle, thick line*) and MS supplemented with 1.5 mg/l of 2,4-D (*empty square*, *dashed line*)

In Fig. 3, it is possible to observe the growth curves for the cell suspensions in the treatments of MS without promoters and MS supplemented with 1.5 mg/l of 2,4-D. It shows that the MS medium without promoters presents a lag phase of approximately 7 days, followed by an exponential phase that extends during 23 days, reaching values of up to 3.0 × 10^4^ cells/ml. In regards to the growth curve for the MS medium with 1.5 mg/l of 2,4-D, the lag phase is longer, of 10 days, and the exponential phase lasts 20 days, with cell concentrations of 2.4 × 10^4^ cells/ml. There is a considerable similarity between both plant cell suspensions, with MS medium without promoters being the treatment that presented the best results for the blackberry plant cell suspension growth.

Additionally, the total polyphenol quantification determined that the MS treatment presented equivalent 0.0256 mg of gallic acid/g of fresh sample, with a cell concentration of 1.0 × 10^6^ cells/ml.

### Cell analysis through OM and TEM

**Fig. 4:**
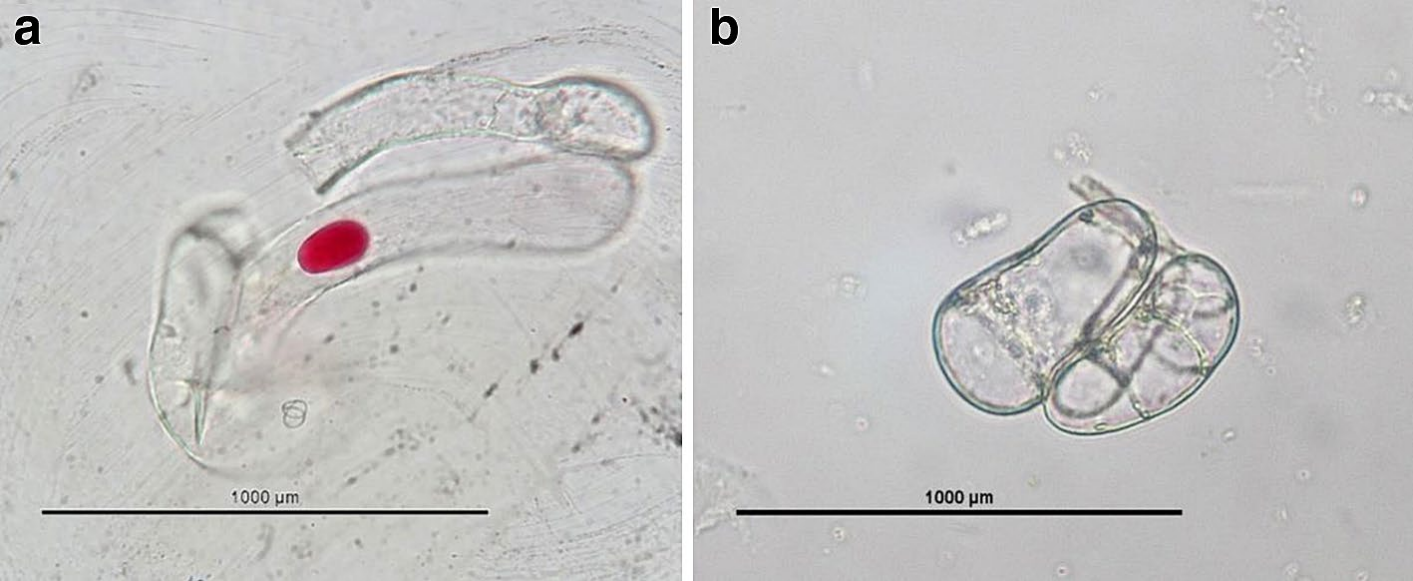
Blackberry cells viewed under an optical microscope, obtained from callus containing polyphenols (**a**) and from cell suspensions (**b**)

The calli, being influenced by the light, presented a coloration change from a yellowish tonality to purple, and when observed under the OM it was possible to visualize the colored content inside the plastids (Fig. 4a). On the other hand, the sample from the best treatment of cell suspensions can be seen in Fig. 4b, where the cells are small and rounded, which demonstrates that they are under a stage of cell growth.

**Fig. 5:**
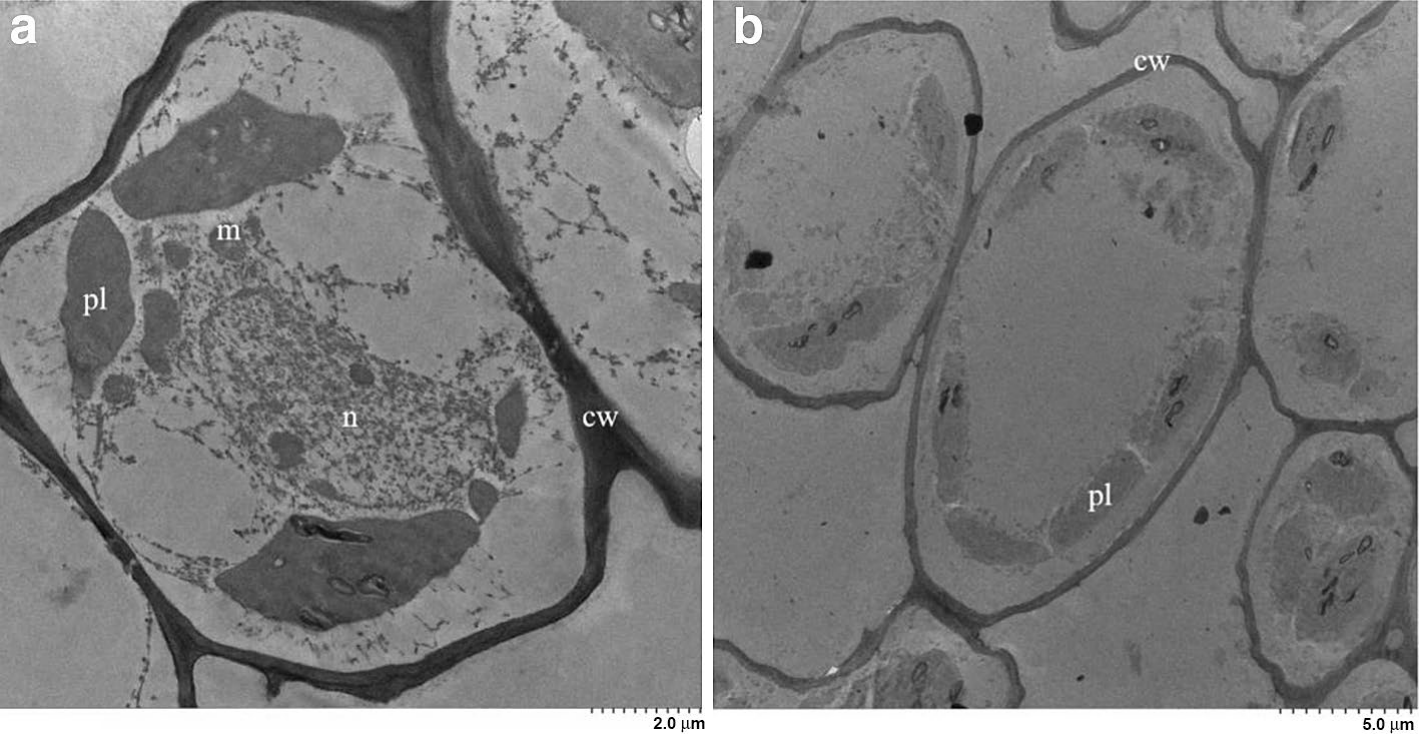
Blackberry cells observed through TEM and obtained from the best callogenesis treatment (**a**) and from a cell suspension (**b**). In figure, nucleus (n), cellular wall (cw), mitochondria (m) and plastids (pl) was observed

Figure 5 shows the callus cells obtained from the MS medium with 1.5 mg/l of 2.4-D and the cell suspension in MS medium with no growth regulators. It is possible to observe more differentiation in terms of histological development in callus cells, when compared to the cell sample from cell suspensions. The blackberry cells from the cell suspensions present a reduced development of the organelles, a thinner cell wall as well as smaller and rounder cells. In both samples, it is possible to see the presence of a large number of plastids.

## Discussion

### Callogenesis induction

The techniques for plant biotechnology related to the production of compounds of interest, include the growth of plant cells or organ culture in bioreactors (Verpoorte and ten Hoopen 2006). In order to achieve the bioreactor upstream process, it is necessary to standardize the previous stages, corresponding to callus production and the establishment of cell suspensions.

The first stage consists in the development of the callus culture, which is obtained from the growth and maintenance of an unorganized cell mass, formed from segments of tissue, organs or cells that have been previously cultured (Chattopadhyay et al. 2002; George 2008). Under the stimulus of endogenous or chemical growth promoters added to the culture medium, the plant cell metabolism that remained in a quiescent state, is activated and leads to the start of cell division. During this process, the differentiation and cell specialization, which could have occurred in the plant, is reversed and the explant generates a new tissues comprised of meristem and non-specialized cells (George 2008). In this study, leaves from the second and third position in the plagiotropic axis (the younger leaves) were selected, due to the fact that they generate more friable callus (Hollmann et al. 2002).

Generally, auxins are required for the callus induction from plant explants. When they are applied to the culture medium, they have the ability to alter the programmed physiology in the complete tissue of the plant. The cells that respond to the auxin are reverted to a dedifferentiation stage and start to divide (George 2008). For the callus formation, different concentrations of 2,4-D were used, since according to González et al. (2003), the auxins produce elongation and formation of adventitious roots at low concentrations, while at higher concentrations, they induce callus formation, being 2,4-D the most frequently used auxin (George 2008).

In a study developed by Hollmann et al. (2002), callogenesis was induced from leaves of three different species of *Rubus* spp. Three different semi-solid culture media were tested, Anderson, Heller and MS, supplemented with 2 % sucrose, vitamins and agar. The growth promoters used for the callogenesis were 2,4-D; picloram, naphthaleneacetic acid (NAA), indolebutyric acid (IBA), kinetin, benciladenin (BA), 2-isopentenyladenin (2-ip) and coconut water; incubating the explant under dark conditions. For *R. parviflorus* the medium containing 4.52 µM of 2,4-D was the best treatment, while for the blackberry species *R. spectabilis* and *R. strigosus*, the media supplemented with 26.9 µM of NAA and 12.4 µM of picloram, respectively, allowed to obtain the higher percentages of callus formation and robustness.

On the other hand, Cruz et al. (2009) produced plant callus from leaves of *Rubus adenotrichos* Schltdl., using as growth promoters BA, IBA, NAA and kinetin in concentrations ranging between 0.5 and 2.0 mg/l, incubating the explants at 22 ± 2 °C with a 12 h light and 12 h dark photoperiod, where the best results were obtained using NAA and kinetin growth promoters. In the research performed by Mezzetti et al. (1997), the effect of the growth regulators was evaluated in two species *Rubus* sp. From the regulators applied, the medium containing 5 µM of 2,4-D or 10 µM IBA, were the ones which, in all cases, promoted a higher callus production from leave explants.

In this study, the use of 2,4-D produced friable callus in the second subculture, with 1.5 mg/L being the optimal concentration, since it presented the highest median in regards to fresh and dry weight. Related to this, the other treatments with statistically equal results (2.0; 2.5 and 3.0 mg/l of 2,4-D) required a higher concentration of this auxin, which implies a greater chance of somaclonal variation as well as a higher cost.

A callus originated from a new explant or a segment that has been previously established in an in vitro culture, has three development stages: cell division induction, a period of active cell division during which differentiated cells lose any specialization and, finally, a period when cell division is reduced and in the callus there is an increase in cell differentiation. These stages are similar to those found in the growth of a cell suspension on a specific volume of culture medium (batch culture); where, according to the variation in the different parameters that are applied to measure growth, a sigmoidal growth curve is generally obtained. The phases in this curve correspond to the lag, exponential and lineal phases, followed by a declining period and a stationary phase, when the growth stops (González et al. 2011). This sigmoidal growth behavior was observed in callus growth curves obtained in this research, for the variables of fresh as well as dry weight.

### Establishment of the cell suspensions and total polyphenol quantification

The callus can be used for the extraction of compounds of interest, however, the cell suspensions present a greater potential for industrial applications (Chattopadhyay et al. 2002). A cell suspension is a cell population or small clusters of cells growing in an agitated culture medium (George 2008). The development of the cell suspensions is related with the type of explant, culture medium, environmental conditions, agitation speed and cell density.

Borejsza-Wysocki and Hrazdin (1994) established cell suspensions from calli of *Rubus ideaus* cv. Royalty. The callus derived from juvenile leave segments, using two types of basal culture media (MS and Anderson) as well as 2,4-D (9 µM), IBA (4.9 µM) and 2-isopentenyladenin (2iP) (4.9 µM), and the same media was used in liquid state for the establishment of the cell suspensions.

In a research study lead by Hollmann et al. (2002), the cell suspensions were generated from 0.5 to 1.0 g (fresh weight) of callus segments from three species of *Rubus* in 125 ml erlenmeyer flasks containing 25 ml of MS culture medium, supplemented with sucrose and vitamins, and a 150 rpm agitation at 23 °C. Different growth promoters were tested (2,4-D, picloram, NAA, IBA, KIN, BAP, 2iP) regarding their ability to maintain the cell culture growth, obtaining the best results in *R. parviflorus* and *R. spectabilis* with 26.9 µM of NAA, and in *R. strigosus* with 26.9 µM of NAA and 12.4 µM of picloram.

In the current study, the culture medium used was MS culture medium free of growth promoters as well as MS supplemented with 1.5 mg/l of 2,4-D, which was the optimal culture medium for the callus production in semisolid medium, using the same principle as Borejsza-Wysocki and Hrazdin (1994), who, for the establishment of the cell suspensions used the same media as for the callogenesis induction as well as MS without promoters.

Considering that the aim of the study was to obtain a higher growth for cell suspensions rather than callus production a basic MS medium lacking growth promoters represented the most convenient option for the establishment of the cell suspensions. It must also be considered, from a costs point of view, that the option of a simple culture medium is cheaper; also, a growth regulator such as 2,4-D leads to a higher probability of mutations and somaclonal variations. In fact, the MS medium free of growth regulators, resulted in better blackberry cell suspension culture than the one containing 1.5 mg/l of 2,4-D. On the other hand, it was also possible to determine that the adequate agitation for the callus desegregation was of 100 rpm, without causing hydrodynamic stress processes, due to their high friability,

The cell suspension growth curve follows a sigmoidal curve similar to the one obtained for the callus growth (George 2008), where it was possible to observe a lag, exponential and stationary phase. This allows to distinguish which culture media leads to an optimal cell growth, with a greater quantity of biomass during the time of culture; particularly for this study, it corresponded to the MS culture medium.

In a study performed by Acosta-Montoya et al. (2010), a total polyphenol concentration of 4.6 and 5.8 equivalent mg of gallic acid/g of fresh sample was quantified, which is a considerably higher concentration that the one obtained in this research. Hence, for further research in this topic, it is recommended to increase the cell biomass and use elicitors which enhance the total polyphenol and antioxidant concentration.

### Cell analysis through OM and TEM

According to Verpoorte et al. (2002), the in vitro culture of un-differentiated cells present higher growth rates than those from differentiated cells, making the mass transfer processes more efficient, and allowing higher cell density cultures. In the process of un-differentiation, the cells can silence the expression of certain organelles and other structures that can cause unnecessary energy loss, reason for which the cells reduce their size in order to allow a closer communication between the protoplasm of the cells and the rest of the cytoplasm structures (Kino et al. 2001). This modification achieves an enhanced efficiency in the use of nutrients and energy, which according to Samanani and Facchini (2006) promotes the production of secondary metabolites.

Therefore, in the process of un-differentiation observed in the blackberry cells, it was possible to observe important differences in terms of size, shape and cell content when comparing the cells of the callus and the cell suspension; since the cells from the suspension should be smaller and with a thin cell wall in order to reduce the mechanical stress caused by agitation (Meyer et al. 2002). On the other hand, the most common organelles found in the cells in suspension were plastids, being energy storage structures found in plant cells, used in the stationary phase, which is the stage of growth kinetics where cell metabolism is reduced. Additionally, many of the enzymes responsible for the biosynthesis of secondary metabolites can be found in these organelles (Samanani and Facchini 2006).

Finally, the functional compounds stored in the calli cell plastids possibly correspond to phenolic compounds of flavonoid type, due to their coloration and the organelle that stores them (Fernández et al. 2002). These compounds were also obtained in the phenolic compound quantification performed to the cell suspensions. According to Martínez-Flores et al. (2002) flavonoids are natural pigments present in fruits, vegetables, seeds and flowers that protect the tissue from the damage produced by oxidant agents such as UV rays, environmental pollutants and chemical substances that can be present in the plants. Their antioxidant properties are directed towards the capture of superoxide and hydroxide radicals, which are highly reactive species that can be found in the start of the lipid peroxidation process. Additionally, they respond to light and control the auxin level which regulate the growth and plant differentiation. Other functions include their antifungal and bactericide role, the coloration change that contribute to pollinizing phenomena, and their ability to fix metals such as iron and copper.

## Conclusions

The culture medium containing 1.5 mg/l de 2,4-D presented a higher percentage of plant callus formation, as well as a larger sized calli, as per the fresh and dry weight determinations performed. Furthermore, the callus growth curve was of 42 days, while the cell suspension in the MS medium was the treatment that presented a greater growth after 39 days of culture, presenting an equivalent of 0.0256 mg of gallic acid/g of fresh sample. Through this research, it was possible to standardize the callogenesis and cell suspension protocols for blackberry plants (*Rubus adenotrichos* Schltdl.) and obtain its corresponding growth curves, as well as the comparison of its cell differentiation, which will allow to have a better understanding of these processes for the upstream process and large scale production of antioxidants. Additionally, the results obtained evidence the need to continue research associated to this topic, in order to increase the cell suspension biomass concentration, as well as to perform other elicitation and quantification assays, not only for total polyphenols, but also for other functional, specific compounds, such as antioxidants, obtained through different biotechnological techniques.
